# Supplementary material for: Splice-Junction-Based Mapping of Alternative Isoforms in the Human Proteome
Source: Cell Rep. Author manuscript; Available in PMC 2020 Jan 15. (PMC6961840; doi:10.1016/j.celrep.2019.11.026)
Supplement: 2 [file NIHMS1546469-supplement-2.zip › DF1/PXD006675/CardiacFibroblast/CardiacFibroblast_1_PCBP2_YSTGSDSASFPHTTPSMCLNPDLEGPPLELTK.pdf]

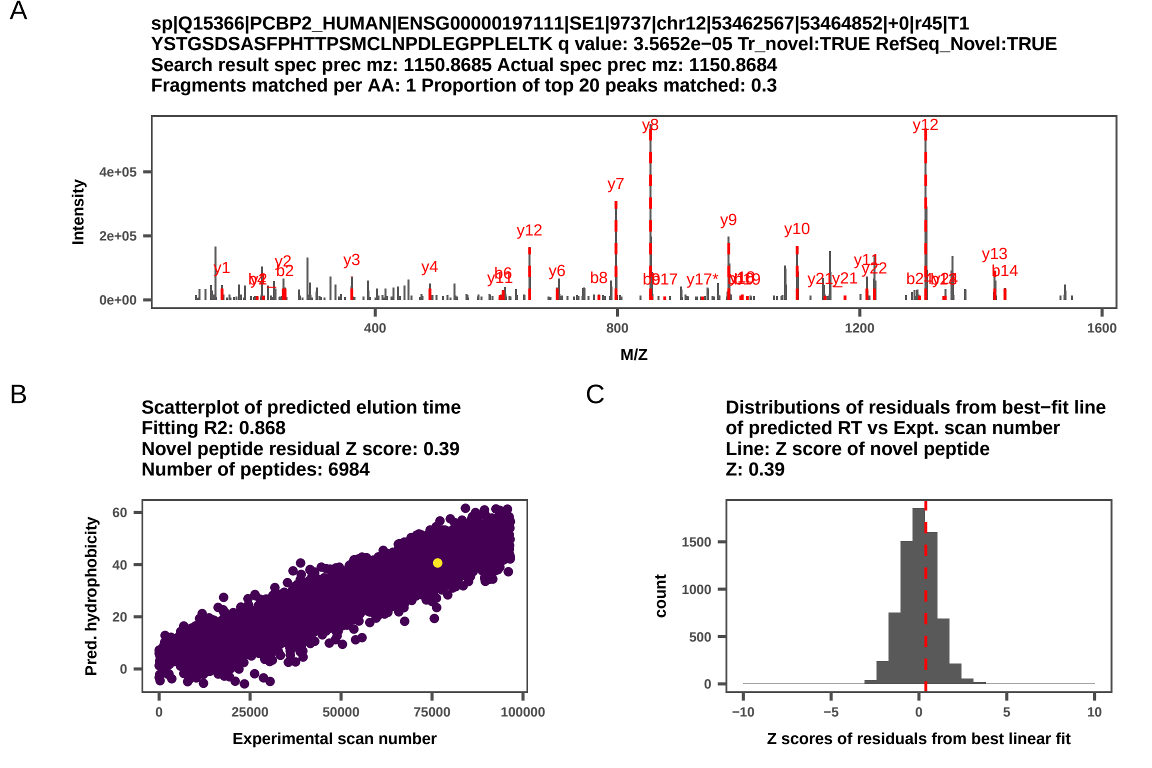
A
sp|Q15366|PCBP2_HUMAN|ENSG00000197111|SE1|9737|chr12|53462567|53464852|+0|r45|T1
YSTGSDSASFPHTTPSMCLNPDLEGPPLELTK q value: 3.5652e−05 Tr_novel:TRUE RefSeq_Novel:TRUE
Search result spec prec mz: 1150.8685 Actual spec prec mz: 1150.8684
Fragments matched per AA: 1 Proportion of top 20 peaks matched: 0.3
Intensity
4e+05
2e+05
0e+00
M/Z
400
800
1200
1600
y1
y2
b2
y3
y4
b6
y12
y6
b8
y7
y8
y9
y10
y12
y13
b14
y17*
B
Scatterplot of predicted elution time
Fitting R2: 0.868
Novel peptide residual Z score: 0.39
Number of peptides: 6984
Pred. hydrophobicity
60
40
20
0
0
25000
50000
75000
100000
Experimental scan number
C
Distributions of residuals from best−fit line
of predicted RT vs Expt. scan number
Line: Z score of novel peptide
Z: 0.39
count
1500
1000
500
0
−10
−5
0
5
10
Z scores of residuals from best linear fit
